# Percutaneous thrombin injection with intra-arterial balloon protection for iatrogenic puncture site pseudoaneurysm: a case series

**DOI:** 10.3389/fradi.2026.1695420

**Published:** 2026-02-11

**Authors:** Chiu-Shih Cheng, Chang-Hsien Ou, Wan-Ching Lin, Ho-Fai Wong

**Affiliations:** 1 Division of Neuroradiology, Department of Medical Imaging, E-Da Hospital, I-Shou University, Kaohsiung, Taiwan; 2 Division of Neuroradiology, Department of Medical Imaging and Intervention, Chang Gung Memorial Hospital, Taoyuan, Taiwan

**Keywords:** balloon, injection, pseudoaneurysm, thrombin, ultrasound

## Abstract

**Objective:**

To evaluate the technical feasibility and preliminary outcomes of percutaneous thrombin injection with intra-arterial balloon protection to treat iatrogenic puncture site pseudoaneurysm (IPA) following neurovascular intervention.

**Methods:**

The data of eight patients treated for IPAs following neurointerventions at a single institution between 2021 and 2024 were retrospectively reviewed. Three, two, and three patients were treated with ultrasound-guided thrombin injection (UGTI), covered stents, and thrombin injection with intra-arterial balloon protection, respectively. The patients treated with thrombin injection with intra-arterial balloon protection were included in the analysis. Two of these IPA cases involved the femoral artery, and one involved the brachial artery.

**Results:**

Technical success, defined as complete thrombosis of the pseudoaneurysm sac with preservation of the parent artery, was achieved in all cases. No procedure-related complications, such as distal embolism or parent artery occlusion, were observed. Antiplatelet therapy was continued without interruption, and the technique remained effective in one patient with mild thrombocytopenia. Follow-up ultrasonography at one day and one week confirmed the absence of recurrence and maintained patency of the parent artery. Furthermore, no symptoms of limb ischemia were observed immediately, one-day, three days, one-week and one-month clinical follow-up period.

**Conclusion:**

Percutaneous thrombin injection combined with intra-arterial balloon protection is a technically feasible and potentially safe method for managing IPAs, particularly in patients requiring uninterrupted antiplatelet therapy. Use of the distal radial approach for balloon access may further reduce puncture-site complications. Larger, prospective studies are warranted to validate these findings and to compare this technique with established treatment modalities.

## Introduction

Iatrogenic puncture site pseudoaneurysms (IPAs) are uncommon complications, occurring in 1.1% to 8% of endovascular procedures (1–3). A higher-than-average incidence of IPAs has been observed in patients administered potent antiplatelet agents during and after neurointerventions. IPAs are associated with significant morbidity and mortality, as they may lead to life-threatening rupture or limb-threatening distal ischemia (4). Although surgical repair is the traditional standard treatment for IPAs that do not resolve spontaneously, this approach has been associated with relatively high morbidity rates of up to 21% (5). Thus, minimally invasive methods have become preferred alternatives, with these including ultrasound-guided compression and percutaneous ultrasound-guided thrombin injection. However, the effectiveness of these methods is limited when compression of the aneurysmal neck is technically unfeasible. Furthermore, there is a risk of thromboembolism due to thrombin leakage or thrombus migration into the parent artery (6). Percutaneous thrombin injection with balloon protection has been proposed as a safer alternative that may minimize the risk of distal thromboembolism (7, 8). However, studies have provided limited data on the safety and efficacy of this combined approach in patients with IPAs who also present with thrombocytopenia and are receiving antiplatelet therapy. We therefore report our preliminary experience regarding the technical feasibility and safety of percutaneous thrombin injection with balloon protection in three patients with IPA following neurointerventions without stoppage of antiplatelet therapy.

## Materials and methods

Eight patients presented with iatrogenic pseudoaneurysms at the puncture site between 2021 and 2024 following endovascular procedures—specifically diagnostic angiography, mechanical thrombectomy, flow-diverter placement, and aneurysm embolization. The selection of the treatment approach was guided by an evolving institutional protocol and increasing technical experience that shifted toward safer method. Earlier cases were managed with established methods, including ultrasound-guided thrombin injection (UGTI; *n* = 3) and covered stents (*n* = 2). As our protocol shifted toward more controlled and safer technique, subsequent cases were selectively treated with thrombin injection combined with intra-arterial balloon (*n* = 3). In addition to clinical considerations, the choice of therapy was influenced by patient-specific financial factors.

This study was designed as a focused case series specifically evaluating the feasibility, safety, and technical outcomes of thrombin injection combined with intra-arterial balloon protection. Therefore, only the three patients treated with this technique were included in the main analysis. Among these patients, two had femoral artery pseudoaneurysms and one had a brachial artery pseudoaneurysm. Patient demographics, causes of pseudoaneurysms, are summarized in Table 1. IPA characteristics, treatment approaches and related details are provided in Table 2. For transparency, the clinical characteristics of the excluded five patients are provided in the Supplementary Table S1.

**Table 1:** Summary of cases.

Case	Sex/Age	Location of IPA	Catheter size	Related procedure	Hemostatic method	Onset of symptoms	Ongoing antiplatelet therapy	PLT, PT, INR
1	F/88	Right superficial femoral artery	9Fr.	Mechanical thrombectomy	Angioseal^TM^	9days	Aspirin100 mg	PLT: 477 × 10^3^/μL PT: 11.1 s, INR: 1.08
2	F/62	Right common femoral artery	5Fr.	Diagnostic cerebral angiography	Manual compression	1day	Aspirin100mg	PLT: 162 × 10^3^/μL PT: 11 s, INR: 1.1
3	M/70	Right brachial artery	7Fr.	Carotid stenting	Angioseal^TM^	1day	Aspirin100 mg + Clopidrogrel75 mg	PLT: 124 × 10^3^/μL PT: 10.6 s, INR: 1.02

F, female; Fr., French; INR, international normalized ratio; IPA, iatrogenic puncture site pseudoaneurysm; M, male; mg, milligram; μL, microliter; PLT, platelet count; PT, prothrombin time; sec, second; U, unit.

**Table 2:** Summary of pseudoaneurysm characteristics and treatment procedure.

Case	Location	Size of IPA	Neck of IPA (diameter x length)	Dose of thrombin	Number of injection	Balloon access site	Total balloon inflation time	Fluoroscopy time	Procedure time
1	Right superficial femoral artery	2.8 × 1.9 cm	Not well defined neck	1,000 U	1time	Left common femoral artery	5 min	3.5 min	30 min
2	Right common femoral artery	1 × 0.5 cm	3 × 1 mm	4,000 U	4times (1,000 U each)	Left distal radial artery	20 min	5 min	50 min
3	Right brachial artery	2.3 × 2.1 cm	4 × 4 mm	1,500 U	2 times (1,000 U + 500 U)	Right distal radial artery	10 min	2 min	40 min

cm, centimeter; IPA, iatrogenic puncture-site pseudoaneurysm; mm, millimeter; min, minute; U, unit.

### Ethical considerations and statistical analysis

Due to the retrospective, observational nature of the study, institutional review board (IRB) approval was waived. No formal statistical analysis was performed due to the descriptive nature of this case series and the small sample size (*n* = 3). All data are presented descriptively.

### Procedure

**Figure 1:**
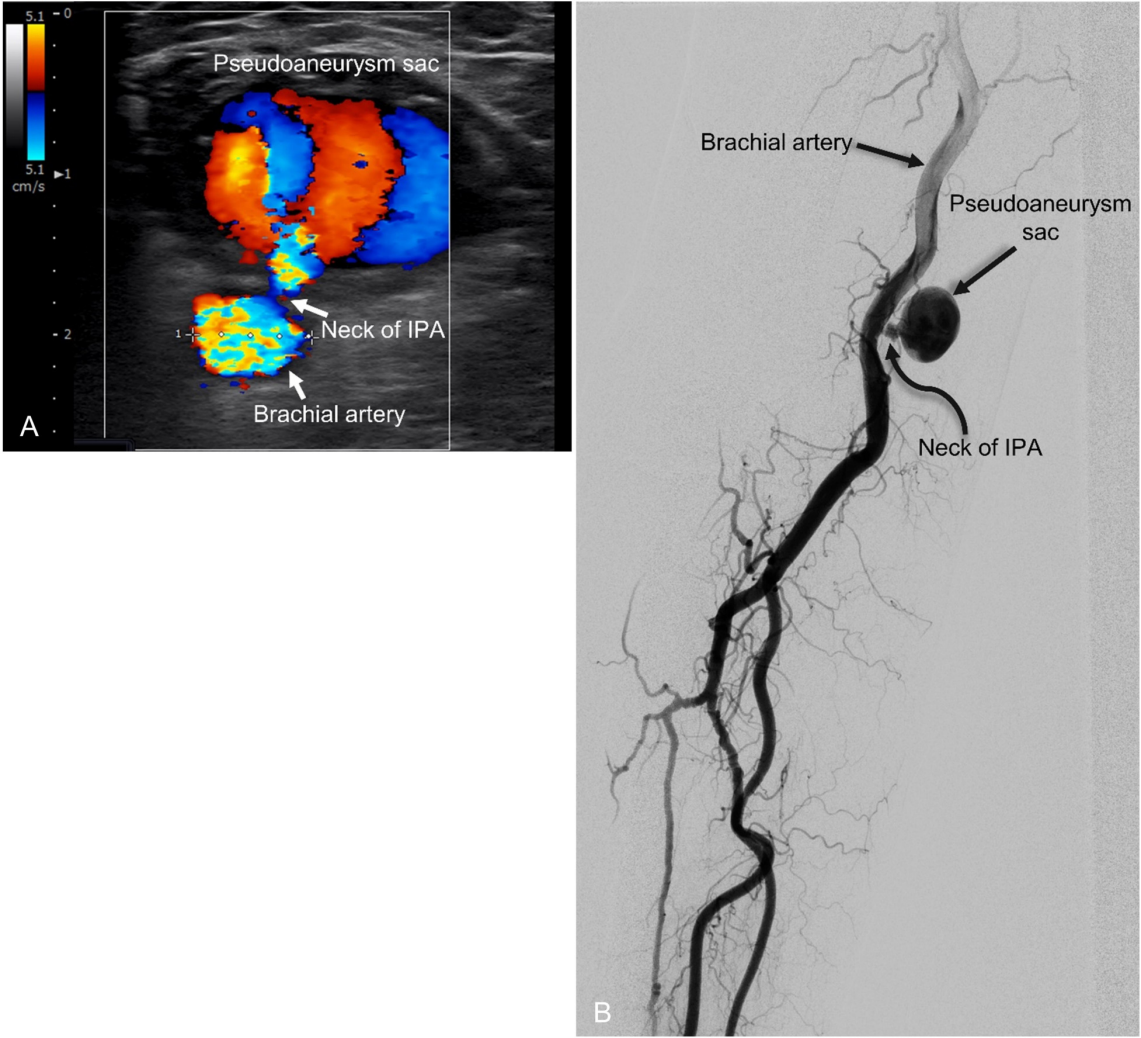
Ultrasound and angiographic images of a representative case of brachial IPA (case 3). **(A)** Doppler ultrasound image of brachial puncture site pseudoaneurysm in the transverse plane, revealing internal flow and connection to the brachial artery. **(B)** Angiography revealed the pseudoaneurysm connection to the brachial artery.

**Figure 2:**
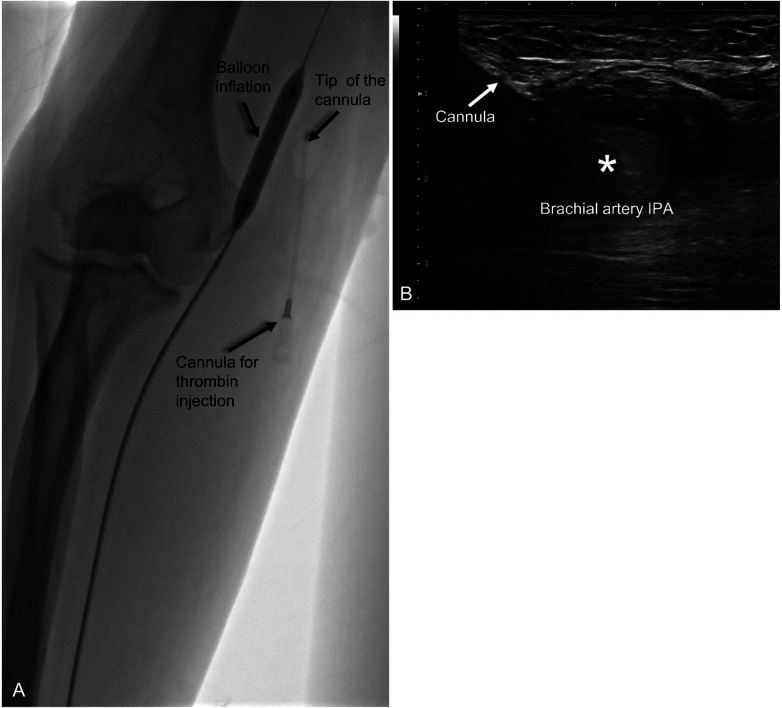
Ultrasound and angiographic images of percutaneous thrombin injection with intra-arterial balloon protection procedure. **(A)** Angiography showing balloon inflated in the right brachial artery across the pseudoaneurysm neck through the right distal radial artery approach. Thrombin was injected through a cannula inserted into the pseudoaneurysm with tip of the cannula placed at the center of pseudoaneurysm. **(B)** Sonography of brachial pseudoaneurysm with the cannula (arrow) indicated within the pseudoaneurysm sac. Hyperechoic portion (asterisk) within the sac indicates thrombus formation following thrombin injection.

All procedures were performed under local anesthesia. Initial assessment was conducted using Doppler ultrasound (DUS) (SONIMAGE HS2, Konica Minolta, Tokyo, Japan) with a L18-4 linear transducer (4–18 Mhz) to confirm the presence and morphology of IPA (Figure 1A). Vascular access for balloon protection was obtained via a 6 Fr sheath through the contralateral femoral artery (Case 1), left distal radial artery (Case 2), and ipsilateral distal radial artery (Case 3). Angiography was performed to confirm the presence of pseudoaneurysm and evaluate the diameter of the parent artery for balloon size selection (Figure 1B). A 7 × 40 mm balloon catheter (Sterling^TM^ Monorail PTA Balloon Dilatation Catheter, Boston Scientific Corporation, US) was used in all cases, with the balloon positioned across the pseudoaneurysm neck (Figure 2A).

**Figure 3:**
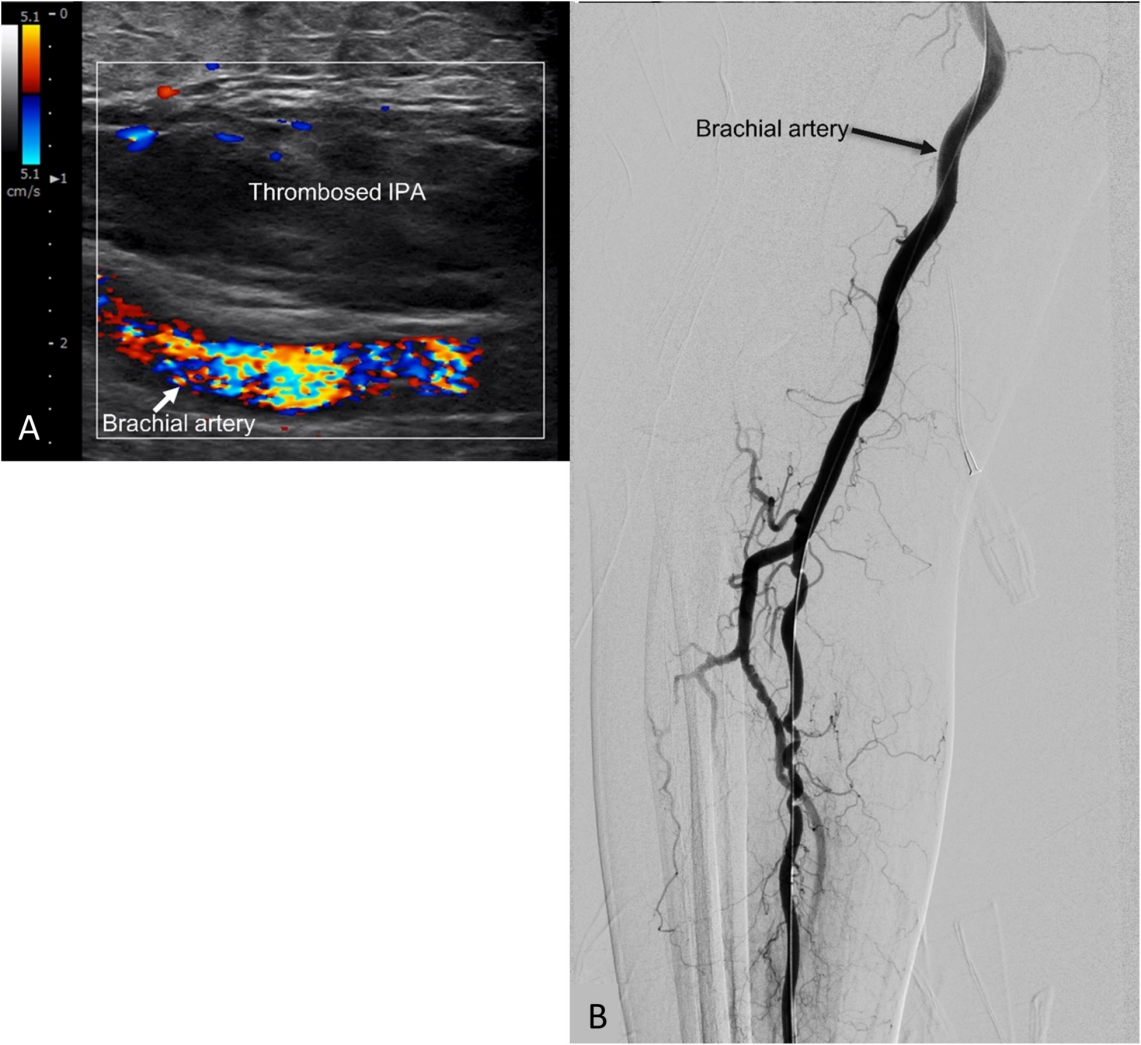
Post-procedure angiography and sonography findings. **(A)** Final sonogram demonstrated complete pseudoaneurysm thrombosis with no internal flow. **(B)** Final angiogram indicated no further contrast filling in the pseudoaneurysm.

Under ultrasound guidance using in-plane technique, the pseudoaneurysm was punctured with an 18-gauge cannula (Surflo^TM^ I.V Catheter, Terumo, Tokyo, Japan). The cannula tip was positioned at the center of the pseudoaneurysm sac, away from the neck. With the balloon inflated to prevent systemic thrombin migration, an initial dose of 1,000 units of thrombin (FloSeal, Baxter Healthcare, Hayward, CA, USA) was slowly injected into the sac over one minute. This initial dosage was selected based on commonly reported literature and institutional experience. During the injection, intraluminal clot formation was visualized in real-time as an increasing hyperechoic area on sonography (Figure 2B). Balloon inflation was maintained for a total of five minutes to allow for stable thrombus consolidation. After this five-minute period, the balloon was deflated, and the IPA was assessed using color DUS (Figure 3A). If DUS indicated complete thrombosis, the result was further confirmed via angiography to verify the absence of flow in IPA and the patency of the parent artery (Figure 3B). If residual flow was detected, repeat injections were administered: 500 U for a 0.5 cm or less residual flow area or 1,000 U for a residual area larger than 0.5 cm, while maintaining balloon inflation. Following each supplemental injection, balloon inflation was maintained for an additional five minutes, and the assessment was repeated until complete thrombosis of the pseudoaneurysm was achieved.

Hemostasis of the puncture site for balloon access was achieved using an Angioseal^TM^ device (Terumo, Tokyo, Japan) for the case with femoral access and manual compression for the cases with distal radial approach. Serial follow-up color Doppler ultrasound studies were performed at one day and one week postoperatively to assess for pseudoaneurysm recurrence and parent vascular patency. Clinical evaluation of limb ischemia symptoms and hematoma size were performed immediately post-procedure and at intervals of one day, three days, one week, and one month. Notably, all patients continued their antiplatelet therapy before, during, and after the procedure.

### Outcome definitions

#### Technical success

Complete absence of contrast filling within the pseudoaneurysm sac on final angiography and total disappearance of internal flow on color DUS immediately following the procedure.

#### Treatment success

Confirmed thrombosis of the sac and maintained patency of the parent artery on follow-up color DUS at 1 day and 1 week.

#### Clinical success

The absence of limb ischemia, new-onset numbness, or hematoma expansion at the 1-month clinical follow-up.

#### Procedure-Related complications

Any occurrence of distal embolization, parent artery vasospasm, vessel dissection, or access-site complications (e.g., radial hematoma) within 30 days.

## Results

Technical success was achieved in one section in all three patients (3/3). The antiplatelet regimens were maintained without interruption and consisted of aspirin (100 mg/day) in Cases 1 and 2, and dual antiplatelet therapy with aspirin 100 mg/day plus clopidogrel (75 mg/day) in Case 3. The antiplatelet regimen was initiated 7 days prior to pseudoaneurysm development and was continued for 6 months for the patient's underlying vascular condition.

The total thrombin doses administered were 1,000 U in Case 1, 4,000 U in Case 2, and 1,500 U in Case 3. Case 1 achieved complete thrombosis after initial 1,000 U of thrombin.

Case 1: Complete thrombosis was achieved immediately following the initial injection of 1,000 U of thrombin.

Case 2: Following the initial injection, a 1 cm residual flow area was identified on DUS. Consequently, three supplemental injections of 1,000 U each were administered until complete thrombosis was achieved.

Case 3: A small residual flow area of less than 0.5 cm was detected after the initial 1,000 U injection. A single supplemental dose of 500 U was administered, resulting in complete thrombosis of the sac.

The procedure times were 30 min for Case 1, 50 min for Case 2 and 40 min for Case 3, and fluoroscopy times were 3.5 min, 5 min and 2 min respectively. Balloon inflation times were 5 min in Case 1, 20 min in Case 2 and 10 min in Case 3.

No procedure-related complications, including distal embolization to the extremities, vasospasm, or vascular dissection at the balloon inflation site, were observed on angiography. In addition, no complications related to the vascular access used for balloon protection were noted.

Treatment success was achieved in all patients (3/3), as confirmed by color DUS performed on postprocedural day 1 and at 1 week, demonstrating persistent thrombosis of the pseudoaneurysm and patency of the parent artery. All patients achieved clinical success at follow-up evaluation conducted at 1 day, 3 days, 1 week, and 1-month, with no reported limb ischemia or numbness. These findings support the safety and efficacy of percutaneous thrombin injection with intra-arterial balloon protection for the treatment of IPAs following neurointerventions. Notably, this technique was effective even in high-risk patients on dual antiplatelet therapy or with thrombocytopenia (Case 3). All procedural outcomes and patient data are summarized in Table 2.

## Discussion

Although IPAs are infrequent, these complications are potentially life-threatening following neurointerventions, with a reported incidence ranging from 1.1% to 8% (1–3). Numerous factors contribute to this variation in incidence, including puncture technique, use of vascular closure devices, operator experience, and patient-specific conditions (e.g., coagulopathy, long-term anticoagulant therapy, arterial hypertension, and obesity) (3, 9). IPAs are most commonly associated with brachial artery punctures or punctures in the lower portion of the common femoral artery or superficial femoral artery (10). Multiple treatment strategies are available for IPA management, each with distinct advantages and limitations (Table 3).

**Table 3:** Comparison of treatment techniques for iatrogenic puncture site pseudoaneurysms (IPAs).

Treatment technique	Reported technical success rate	Recurrence rate	Major complications	Key advantages	Key limitations
Manual compression or ultrasound-guided compression	72%–90%	Up to 10%–30%	DVT, IPA rupture, vasovagal response, arterial thrombosis, nerve compression (≤3.6%)	Non-invasive, widely available	Painful, time-consuming, ineffective in wide-neck, obese patients, or lesions above inguinal ligament
UGTI (without balloon protection)	91%–100%	3%–9% (mostly within 24 h)	Distal arterial embolization (0.3%–1.5%), parent artery thrombosis	High success, minimally invasive, rapid	Risk of thrombin leakage, embolization, especially with short/wide necks
Balloon-assisted UGTI	∼97%–100% (case series)	Low; usually managed with repeat injection	Rare access-site IPA, transient arterial thrombosis	Reduces thrombin migration, improves safety in high-risk anatomy	Requires arterial access, limited evidence, small case series
Covered stent placement	95%–100%	Low	Stent thrombosis, fracture, migration, access-site IPA	Immediate exclusion of pseudoaneurysm	Large sheath size, joint-related complications, loss of future access, need for long-term antiplatelet therapy
Coil embolization/NBCA injection	85%–100%	Variable	Distal embolization, infection	Effective in selected cases	Operator dependent, risk without parent artery protection
Surgical repair	>95%	Rare	Infection, bleeding, nerve injury	Definitive, effective for infected IPAs	Invasive, higher morbidity, costly

DVT, deep vein thrombosis; IPA, iatrogenic puncture site pseudoaneurysm; NBCA, N-butyl cyanoacrylate; UGTI, ultrasound-guided thrombin injection.

Previously, surgical repair was the primary treatment for puncture site pseudoaneurysms. Since 1991, manual compression or ultrasound probe compression has been widely performed as a first-line treatment for IPAs. However, these methods have an overall success rate ranging from 72% to 90% (11). This technique is ineffective in cases of wide-neck pseudoaneurysms, non-identifiable necks, obesity, or pseudoaneurysms located above the inguinal ligament, where compression is ineffective. Prolonged compression is often required, which can be painful and physically exhausting for both patients and physicians and which demands extended use of ultrasound equipment (11, 12). Excessive compression has been associated with a complications rate of up to 3.6%, with complications encompassing deep vein thrombosis, acute rupture of pseudoaneurysm, vasovagal response due to pain, femoral artery thrombosis, and nerve compression, resulting in limb numbness (11, 13–15).

UGTI has been widely reported in the literature and the effective way to treat IPA, with reported success rates ranging from 91% to 100% (2, 11, 16). It can be performed with or without ultrasound probe compression. Thrombin (factor IIa) is a key enzyme in the coagulation cascade that converts fibrinogen to fibrin and forms stable blood clots. Thrombin is activated from prothrombin by the prothrombinase complex (Factor Xa, Factor Va, calcium, and phospholipids) (17). Topical thrombin has been used to induce surgical hemostasis since the mid-20th century and is available in bovine, human plasma–derived, and recombinant forms (18). Because of its associated risks of allergic reactions and antibody formation, bovine thrombin is no longer recommended, especially in previously exposed patients (19, 20). Recombinant human thrombin is now preferred. In pseudoaneurysm treatment, thrombin alone without gelatin matrix is injected, forming a clot that is resorbed over 6–8 weeks.

Thrombin doses applied in pseudoaneurysm treatment vary widely (50–5,000 U), and no relevant guidelines have been established. Pezzullo et al. reported a correlation between thrombin dose and pseudoaneurysm volume (14), whereas Reeder et al. observed no such correlation with a low-dose regimen (average 192 U) (21). In the present series, an initial dose of 1,000 units was selected as a moderate starting dose within the commonly reported range, allowing effective thrombosis while minimizing the risk of thrombin overflow. This stepwise titration approach allowed for real-time dose adjustments based on specific imaging findings: 500 U was administered for residual flow areas smaller than 0.5 cm, and 1,000 U for areas 0.5 cm or larger. Supplemental doses were only administered under balloon protection. This protocol ensures that the total dose is titrated to the minimum volume required for complete occlusion, rather than relying on a predetermined total dose, thereby maximizing procedural safety.

Direct thrombin injection has a success rate of over 97% and recurrence rates of 3%–9%, with recurrence typically emerging within 24 h and often manageable with repeat injections (2, 6, 22). No significant difference in success rates has been reported between human and bovine thrombin. Recurrence or failure is more likely with ongoing anticoagulation, large arteriotomy site lacerations (> 0.8 mm), or infection (15, 23). Certain cases, such as arteriovenous fistula–related pseudoaneurysms, may require surgery (16).

Standard direct thrombin injection without occlusion of the parent vessel may cause thrombus migration into the extremity artery or thrombin leakage into the parent artery. This leakage can cause thrombosis in the distal limb artery, reducing the effectiveness of thrombin in thrombosing the pseudoaneurysm (2, 16). Although rare (0.3%–1.5%), this complication is limb-threatening and often requires urgent surgical intervention (6, 24, 25). Furthermore, the precise thrombin injection site significantly influences both successful thrombogenesis within the pseudoaneurysm sac and the risk of thromboembolism. *In vitro* thrombogenesis studies by Kim et al. and Kang et al. demonstrated that injecting thrombin into the center of the sac over approximately 8 s—rather than near the neck—optimizes thrombogenesis and reduces the risk of the thrombin being washed into the systemic circulation (26, 27). Even with optimal technique, however, complete elimination of embolic risk may not be achievable.

Balloon-assisted thrombin injection was developed as a modification of standard UGTI to temporarily protect the parent vessel during thrombin delivery (28–30). This modification prevents complications such as thrombus migration and thrombin leakage while ensuring optimal concentration of thrombin within the pseudoaneurysm sac for enhanced therapeutic efficacy. Previously published case reports and case series describing balloon-assisted thrombin injection are summarized in Table 4. Across 17 publications, comprising a total of 62 cases (including the present study), technical success rates were uniformly high. Recurrence was reported in two cases within a 19-patient cohort: one case was attributed to an elevated international normalized ratio (INR) and resolved after correction of the INR without additional intervention, while the other required a second thrombin injection and subsequently resolved (7). Procedure-related complications were rare, with only one reported case of superficial femoral artery thrombosis at the balloon inflation site (7). Notably, employing a distal radial approach through the snuff box, pedal or posterior tibial artery access for balloon placement demonstrated advantages in reducing the risk of puncture site pseudoaneurysm (31, 32).

**Table 4:** Published case series of balloon-assisted thrombin injection for IPAs (including present study).

Study (year)	No. of cases	Age (years)	Related procedure	IPA location	IPA dimensions	Neck characteristics	Thrombin dose (U)	Balloon access site	Balloon inflation time	Success rate	Complication
Elford et al. (1999) (22)	1	83	Central venous catheterization	Axillary A	6 cm	Not reported	1,000	Not reported	Not specified	100%	None
Bhat and Chakraverty (2007) (42)	1	65	Central venous catheterization	SFA	Not reported	Not reported	700	Contralateral femoral A	Seconds	100%	SFA thrombosis at balloon inflation site, resolved by thromboaspiration
Ergun et al. (2016) (43)	1	52	PCI	SFA	3 × 2cm	No visible neck	Not reported	Contralateral femoral A	1 min	100%	None.
Menon et al. (2018) (44)	4	47–65	PCI	2 SFA, 1 CFA	2.9–4 cm	2.2–7.1 mm	1,000 U	Contralateral femoral A	Not specified	100%	None.
Clark and Abraham (2000) (45)	2	41–76	Hemodialysis AVfistula	Brachial A	1.5–2.5 cm	Not reported	500–700 U	Femoral A	1 min	100%	None.
Owen et al. (2000) (7)	19	33–83	Not reported	Femoral A, PTA, popliteal A.	Not reported	Not reported	1,500 U	Femoral A	15 min	100% Clinical Success (2 recurrences: 1 resolved with INR correction; 1 required 2nd injection)	1 access-site IPA
Samal et al. (2001) (46)	4	47–88	Thrombectomy for leg PAOD, TAVR, PCI	Femoral artery	2.5–7 cm	Not reported	Not reported	Contralateral femoral A	Not reported	100%	None.
Holder et al. (2002) (47)	1	71	Venous catherization	CCA	3.5 cm	Not reported	250 U	Femoral A	10 s	100%	None
Vowels et al. (2020) (48)	1	59	Thrombectomy for leg PAOD	SFA	4.1 × 1.2 cm	5 mm	Not reported	Not specified	Not reported	100%	None
Hayakawa et al. (2021) (8)	11	36 ± 10.43	PCI, Hemodialysis, endovascular therapy	Femoral A	24.34 ± 13.54 cm	Not reported	677.3 ± 410	10 femoral A, 1radial A	Not reported	100%	None.
Watanabe et al. (2022) (40)	1	64	IABP puncture site	SFA	1.6 × 1.4 cm	Not specified	1,000	Left radial A	15 min	100%	None
Patel et al. (2023) (29)	1	42	Femoral hemodialysis catheterization	SFA	5.4 × 3.2 × 2.5 cm	2.2 mm	1,250	Contralateral femoral A	13 min	100%	None
Chowdhury et al. (2024) (41)	1	58	EVAR	Profunda femoris A	3.7 × 2.1 × 1.6 cm	Not reported	3,000	Left radial A	Not reported	100%	None.
Bruno et al. (2024) (30)	3	71–82	Not specified	SFA	2.3–3 cm	Tight neck	750–1,500	Contralateral femoral A	3 min	100%	None
Sarwar et al. (2025) (32)	6	53–85	Subclavian stent, PCI, iliac stenting, TAVR, CFA thrombectomy	CFA	1.3–2 cm	6–7 mm	195–360	2 pedal A, 2 PTA, 2 radial A	Not reported	100%	None
Saha et al. (2025) (49)	2	58–74	PCI	CFA	4–4.2 cm	15–20 mm	1,500 U	Contralateral femoral A	Not reported	100%	None
Present study	3	62–88	Neurointerventions	SFA, CFA, brachial A	1–2.8 cm	3–4 mm	1,000–4,000	1 femoral A, 2 distal radial A	5–15 min	100%	None

A, artery; AV, arteriovenous; CCA, common carotid artery; CFA, common femoral artery; cm, centimeter; DAPT, dual antiplatelet therapy; EVAR, endovascular aortic aneurysm repair; EVT, endovascular therapy; HTN, hypertension; IABP, intra-aortic balloon pump; INR, international normalized ratio; IPA, iatrogenic puncture site pseudoaneurysm; min, minute; mm, millimeter; PAOD, peripheral arterial occlusive disease; PCI, percutaneous coronary intervention; PTA, posterior tibial artery; SFA, superficial femoral artery; TVAR, Transcatheter Aortic Valve Replacement.

Covered stenting is a highly effective and safe treatment for puncture site pseudoaneurysms (33, 34). Although we have previously performed this technique, its drawbacks include the need for prolonged antiplatelet therapy after stenting and the limited availability of stent sizes. However, its drawbacks include the need for prolonged antiplatelet therapy, limited stent size availability, and the potential for joint-related complications or deep femoral artery coverage. Future vascular access to the corresponding vessel may also be compromised after stent placement.

Our preliminary experience suggests that percutaneous ultrasound-guided direct thrombin injection with balloon protection is a feasible and safe treatment option for IPAs. Importantly, this technique was successful in high-risk patients receiving uninterrupted dual antiplatelet therapy or with thrombocytopenia, allowing effective pseudoaneurysm management without cessation of antiplatelet therapy. Compared with covered stent placement, balloon-assisted thrombin injection preserves the parent vessel and avoids permanent implants, thereby maintaining future vascular access and reducing the risk of long-term stent-related complications such as fracture or migration. Additionally, balloon protection may reduce thrombin leakage and distal embolization compared with standard UGTI, while also allowing effective treatment of pseudoaneurysms associated with vascular dissection by reapposing the dissection flap against the vessel wall. A potential drawback of this method is the requirement for arterial access to place the balloon, which carries an inherent risk of secondary IPA, especially in patients with coagulopathy or on antiplatelet therapy (7). This risk can be mitigated by employing a distal radial approach (via the “snuffbox”), which is associated with a lower incidence of access-site complications (8, 32, 40, 41). Furthermore, the use of a 5–6 Fr access sheath for balloon placement facilitates easier hemostasis and permits a distal radial (snuffbox) approach, which is often more suitable for the smaller arterial diameters found in Asian populations than the 7–10 Fr sheaths required for covered stents. For femoral IPAs, we recommend the left distal radial approach specifically, as the shorter anatomical distance to the femoral artery optimizes the working length of the balloon catheter. Finally, the procedure is generally well-tolerated under local anesthesia, offering a high success rate with the option for repeat treatment if recurrence occurs (21).

Other endovascular treatments, such as transcatheter or direct puncture coil embolization and N-butyl cyanoacrylate (NBCA) injection, are an effective but limited treatment approach. Their limitations include increased pressure in cavities without a true vessel wall, infection risk, and the need for skilled operators (35, 36). NBCA injection without parent artery protection risks distal embolization due to circular flow in the pseudoaneurysm. Corso et al. successfully applied NBCA with ultrasound compression of the neck, but this method may induce leakage (37). Balloon-protected percutaneous NBCA injection was also described in the literature (38).

Although IPA can be managed with surgical repair, this approach is associated with substantial risks, including infection, bleeding, and damage to adjacent structures (5, 39). Despite its effectiveness, surgery is expensive and carries particular risks for patients with comorbidities. Surgical repair can be particularly effective in infected pseudoaneurysms or hematomas with skin necrosis (2).

Despite the promising nature of these initial results, we acknowledge several limitations to our study. First, the small sample size renders these findings preliminary and limits the generalizability of our results to a broader, more diverse patient population. Second, a degree of selection bias is inherent in our study design; the choice of treatment was influenced by the evolution of our institutional protocol and patient-specific financial considerations, as thrombin injection and protection devices often involve out-of-pocket expenses compared to insurance-covered stenting. Consequently, our cohort may not represent the full clinical spectrum of IPA management. Further prospective studies with larger, multicenter cohorts are warranted to confirm the long-term safety and efficacy of this technique and to compare its outcomes directly with conventional treatment modalities.

## Conclusion

Percutaneous thrombin injection with intra-arterial balloon protection demonstrated high technical success for the management of IPAs in this case series. Balloon protection of the parent artery appears to be a feasible method to potentially reduce complications, such as distal thromboembolism. Furthermore, the distal radial approach represents a favorable option for balloon access. While these preliminary findings suggest the technique is a viable option even in patients on antiplatelet therapy or those with mild thrombocytopenia, the results should be interpreted with caution given the small sample size. Larger prospective studies are required to establish the comparative safety and efficacy of this approach against conventional treatments.

## Data Availability

The original contributions presented in the study are included in the article/Supplementary Material, further inquiries can be directed to the corresponding author.
