# Supplementary material for: Percutaneous thrombin injection with intra-arterial balloon protection for iatrogenic puncture site pseudoaneurysm: a case series
Source: Front Radiol. 2026 Feb 11;6:1695420. doi: 10.3389/fradi.2026.1695420 (PMC12932444; doi:10.3389/fradi.2026.1695420)
Supplement: Supplementary file 1 [file Table1.docx]

Summary of IPA cases

| Case | Sex/  Age | Location, size and neck of IPA | Risk factor | Ongoing Antiplatelet Therapy | PLT, PT, INR | Treatment of IPA, dose of thrombin |
| --- | --- | --- | --- | --- | --- | --- |
| 1 | F/88 | Right superficial femoral artery  2.8x 1.9cm, no well-defined neck | Antiplatelet therapy | Aspirin 100mg | PLT: 477x10^3^/μL  PT: 11.1sec, INR: 1.08 | UGTI with balloon protection  1000U |
| 2 | F/62 | Right common femoral artery  1x 0.5cm, 3x1mm | Antiplatelet therapy | Aspirin 100mg | PLT: 162x10^3^/μL  PT: 11sec, INR: 1.1 | UGTI with balloon protection  4000U |
| 3 | M/70 | Right brachial artery  2.3x 2.1cm, 4x4mm | DAPT, mild thrombocytopenia | Aspirin 100mg+ Clopidrogrel 75mg | PLT: 124x10^3^/μL  PT: 10.6sec, INR: 1.02 | UGTI with balloon protection  1500U |
| 4 | F/72 | Right common femoral A  2.3x 2cm, 3x 2mm | Immune thrombocytopenic purpura | No | PLT: 230 x10^3^/μL  PT: 10.9sec, INR: 1.15 | UGTI without balloon protection  1250U |
| 5 | M/49 | Right common femoral A  1x 0.7cm, no well-defined neck | DAPT | Aspirin 100mg+ Clopidrogrel 75mg | PLT: 162 x10^3^/μL  PT: 10.0sec, INR: 1.1 | UGTI without balloon protection  2500U |
| 6 | M/18 | Right common femoral A  1.2x 1cm, 3x 1mm | Henoch-Schönlein Purpura | No | PLT: 170 x10^3^/μL  PT: 12.0sec, INR: 1.2 | UGTI without balloon protection  1500U |
| 7 | M/80 | Right superficial femoral A  1.3x 0.8cm, no well-defined neck | DAPT, thrombocytopenia, superficial femoral A puncture | Aspirin 100mg+ Clopidrogrel 75mg | PLT: 78 x10^3^/μL  PT: 11.0sec, INR: 1.09 | Covered stent  (Viabahn) |
| 8 | M/76 | Right superficial femoral A  2.1x 1.6cm, 2.4x 1.5mm | DAPT, superficial femoral A puncture | Aspirin100mg+ Clopidrogrel 75mg | PLT: 162 x10^3^/μL  PT: 9.8 sec, INR: 0.94 | Covered stent  (Viabahn) |

IPA, iatrogenic puncture site pseudoaneurysm; PLT, platelet count; PT, Prothrombin Time; INR, International Normalized Ratio; M, male; F, female; cm, centimeter; mm, millimeter; DAPT, dual antiplatelet therapy; mg, milligram; μL, microliter; sec, second; U, unit.

All eight procedures achieved 100% technical success in a single session with no observed complications. While covered stents are covered by national insurance, thrombin injection requires an additional expenditure of 10,000 NTD for the drug.
